# Powerful association test combining rare variant and gene expression using family data from Genetic Analysis Workshop 19

**DOI:** 10.1186/s12919-016-0039-4

**Published:** 2016-10-18

**Authors:** Yen-Yi Ho, Weihua Guan, Michael O’Connell, Saonli Basu

**Affiliations:** Division of Biostatistics, School of Public Health, University of Minnesota, Minneapolis, MN 55455 USA

## Abstract

**Background:**

Genetic association studies aim to test for disease or trait association with genetic variants, either throughout the human genome or in regions of interest. However, for most diseases and traits, the combined effects of associated genetic variants explain only a small proportion of the genetic variation. This “missing heritability” may be a result of the small effects of common variants considered in the genetic association studies. Rare variants may also play an important role in understanding the missing heritability of complex traits.

**Method:**

We propose a novel weight-adjustment approach to combine gene expression into rare variant analysis. Results from previous simulation studies suggested that incorporating gene expression information can lead to substantial gain in statistical power.

**Results:**

Using the family data set provided through the Genetic Analysis Workshop 19, we identified susceptible genes associated with blood pressure regulation.

**Conclusions:**

These findings provide valuable information for further functional studies for blood pressure control and mechanism.

## Background

In the past decade, genome-wide association studies (GWAS) have been successful in identifying susceptible genetic loci for many complex traits [1]. However, the study by Eichler et al. reported that the amount of genetic variations explained by the findings from GWAS for a given disease or complex trait is often notably less than the estimated heritability of the traits [2]. One explanation is that the common variants examined by GWAS often have smaller effects, and the rare variants with larger genetic effects are often excluded in a GWAS analysis.

Rare variants may play an important role in explaining the “missing heritability” of complex traits. As a result of recent advances in high-throughput sequencing technology, it is becoming financially feasible to assay rare genetic variations in thousands of individuals. The rare variants here are defined as genetic variants with a minor allele frequency of less than 1 %. Hence the typical GWAS strategy of analyzing one variant at a time is oftentimes underpowered for rare variant detection unless the effect size of the variant or the sample size is very large. A number of methods have been developed to analyze multiple rare variants jointly [3–5]. In this paper, we consider the Seq-aSum-VS approach developed by Basu and Pan [3]. This approach uses dimension-reduction and data-adaptive variable selection strategies to identify the nonnull variants from a group of genetic variants and uses a score test to test for association between the group of variants and the disease of interest.

It often still requires a relatively large sample size for rare variant analyses. To boost the statistical power of genetic association analysis, one research direction is to integrate various genomic information, such as gene expression, rare variants, copy number variation, methylation, transcriptional regulation, and protein abundance. With the availability of both rare variant genotype and gene expression information in the family data set through Genetic Analysis Workshop 19 (GAW19), we proposed a novel approach to incorporate gene expression into rare variant association analysis in this paper.

Genovese et al. [6] introduced the idea of using prior knowledge to weight *p values* from a genome-wide association study and provided theoretical proof for controlling family-wise error rate (FWER). In this paper, our contribution is to provide a formal mechanism to construct weights using available information about rare variants and gene expression. Studies by Li et al. [7] and Ho et al. [8] demonstrate that by incorporating additional genomic information, the weight-adjustment procedure can increase statistical power drastically compared to the traditional genetic association analysis.

## Methods

There are 259 participants with full genotype, expression, and blood pressure data available in the GAW19 family data set. Our analysis focuses on systolic blood pressure (SBP) and diastolic blood pressure (DBP) as outcomes of interest. The average SBP and DBP from all visits were used as the summary measurement respectively for every participant. To adjust for pedigree structure in this data, we estimated identity-by-descent (IBD) matrix (Σ) using genome-wide single nucleotide polymorphism (SNP) marker data obtained using the Illumina platform provided through GAW19. To incorporate the dependence information embedded in the family structure, we transformed the average blood pressure measurements: $\mathrm{Y}\sim \mathrm{M}\mathrm{V}\mathrm{N}\left(\upmu, \Sigma \right),\kern0.5em {\mathrm{Y}}^{*}={\Sigma}^{-\frac{1}{2}}Y\sim \mathrm{M}\mathrm{V}\mathrm{N}\left(\upmu, \mathrm{I}\right)$, so that individuals from the same family are independent in the transformed phenotypic value (Y^*^). In addition, to account for the population stratifications that exist in the Mexican American population, we performed multidimensional scaling and calculated the first 3 principal components. In the following analysis, the residual from the transformed phenotypic value adjusting for the first 3 principal components were used. We considered genetic variant with a minor allele frequency of less than 0.01 as rare variant, and performed rare variants analysis for the genes reported by hg19 build on the odd-number autosomes that have less than 50 rare variants and more than 1 rare variant.

### Sequential sum test

Consider *k* rare variants in a gene and that *SNP*_*i*_ indicates the number of rare variant alleles in variant *i* in a general regression equation: $g\left(E(Y)\right)={\beta}_0+{\displaystyle {\sum}_{i=1}^k}{\gamma}_iSN{P}_i{\beta}_c$, with *γ*_*i*_ = *ν*_*i*_ *s*_*i*_; where *s*_*i*_ is 1, −1, or 0, indicating whether the effect of rare variant *i* is positive or negative or excluded from the equation, and *v*_*i*_ is a weight assigned to rare variant *i*. In our analysis, we assumed v_i_ = 1. In addition, *β*_*c*_ represents the common odds ratio between the trait and the rare variants in the gene. We performed the Seq-aSum-VS approach described in Basu and Pan [3] and obtained *p* value for each gene with 500 permutations.

### Constructing weights using expression measurements

After obtaining the *p* value for each gene from the Seq-aSum-VS test, we used gene expression information to construct weight for each gene. In Roeder et al. [9, 10], the authors suggested to use a weight (*w*_*i*_ > 0) to adjust *p* value (*p*_*i*_) and to reject the null hypothesis if it belongs to the set of all gene *i* for which *p*_*i*_/*w*_*i*_ ≤ α. The weight adjustment procedure maintains the proper FWER control as long as *w*_*i*_ > 0 and ${\overline{w}}_i=1$.

Building on the theoretical findings, we developed a novel weight-adjustment approach for rare variant association analysis. After weight adjustment, genes that have strong contributions to phenotype-associated gene expression will be assigned weights greater than 1, hence achieving smaller weight-adjusted *p* values. The weighting mechanism is as follows: we assign a weight *w*_*i*_ to each gene and the weight is the product of 2 parts: $$ {w}_{g_i{E}_j} $$ and $$ {w}_{E_jP} $$. The first term$$ ,\ {w}_{g_i{E}_j}, $$indicates the effect of gene *g*_*i*_ on the *j* ^th^ gene expression measurement, *E*_*j*_. The second term, $$ {w}_{E_jP}, $$ describes whether gene expression measurement (*E*_*j*_) is associated with the phenotypic outcome *(P)*. Eq. (1) is applied to obtain $$ {w}_{g_i{E}_j} $$:

1 $$ {E}_j={\beta}_{0_{ij}}+{\beta}_{g_i{E}_j}\times {g}_i+{\gamma}_{ij}\times P+{\epsilon}_{ij} $$

and ${w}_{g_i{E}_j}={\left(\frac{\widehat{\beta_{g_i{E}_j}}}{\mathrm{SE}\left(\widehat{\beta_{g_i{E}_j}}\right)}\right)}^2$. In equation 1, *g*_*i*_ is the number of total rare variants in gene *i* calculated by collapsing the genotypes across rare variant loci. A second equation (2) was implemented to obtain ${w}_{E_jP}={\left(\frac{\widehat{\beta_{E_{jP}}}}{\mathrm{SE}\left(\widehat{\beta_{E_jP}}\right)}\right)}^2$:

2 $$ P={\beta}_{0_{ij}}+{\beta}_{E_jP}\times {E}_j+{\gamma}_{ij}\times {g}_i+{\epsilon}_{ij} $$

The benefit of taking the product of 2 weights is that if either ${w}_{g_i{E}_j}$ or ${w}_{E_jP}$ is zero, then the resulting product will be zero. On the other hand, if both ${w}_{g_i{E}_j}$ and ${w}_{E_jP}$ are substantially large, then taking the product of the two parts will result in an amplified overall weight. In other words, if rare variants in the gene under consideration provide a strong contribution to outcome *P* through *E*_*j*_, then ${w}_{g_i{E}_j}\times {w}_{E_jP}$ will be a large value.

A crude weight for gene *i* is set to be the maximum of the products among all gene expression measurements: ${w}_{M{P}_i}={\mathrm{Max}}_j\left({w}_{g_i{E}_j}\times {w}_{E_jP}\right)$. To ensure that ${\overline{w}}^{*}=1$, we divide crude weights (${w}_{M{P}_i}\Big)$ by their average ($\overline{{\mathrm{w}}_{\mathrm{MP}}}$) as required by Roeder and Wasserman [10]: ${w}_{M{P}_i}^{*}=\frac{w_{M{P}_i}}{\overline{w_{M{P}_i}}}$. If ${w}_{M{P}_i}$ is larger than the average, then ${w}_{M{P}_i}^{*}$ will be greater than 1 after dividing by the average. We calculate adjusted *p* value for the *i*th gene as: adjusted *p value* for gene $i=\frac{p\ \mathrm{value}\ \mathrm{of}\ \mathrm{gene}\ i\ \mathrm{reported}\ \mathrm{b}\mathrm{y}\ \mathrm{S}\mathrm{e}\mathrm{q}\hbox{-} \mathrm{aSum}\hbox{-} \mathrm{V}\mathrm{S}\ \mathrm{test}\ }{w_{M{P}_i}^{*}}$. If after adjustment, *p value* becomes greater than 1, then it is set to 1.

For the genes with adjusted *p* value of less than 0.05, we performed gene set enrichment analysis using biological process categories defined in gene ontology (GO). To account for the hierarchical structure in GO terms, we implemented conditional hypergeometric test [11].

## Results

Of the 13,711 genes on the odd-numbered autosomes based on the hg19 build, we considered 6118 genes with less than 50 rare variants and more than 1 rare variant in the analysis. We identified 153 genes with weight-adjusted *p* values of less than 0.05 for SBP or DBP; the top 20 genes are listed in Table 1. The genes with strong contribution to phenotype-associated gene expression levels are assigned weights greater than 1. In Table 1, 17 genes have weights for SBP greater than 1 and 18 genes have weights for DBP greater than 1, indicating that these genes contribute to alterations of phenotype-associated gene expression levels.

**Table 1:** Top 20 genes with adjusted *p* value of <0.05 from the Seq-aSum-VS test for either SNP or DBP

	Gene	Chr	# RVs	*p* Value_*s*_	*W*_*s*_	*p*_s_^*^	*p* Value_*D*_	*W*_*D*_	*p*_D_^*^
1	*NAIF1*	9	23	<0.002	1.306	<0.002	0.002	0.825	0.002
2	*SPATA13-AS1*	13	7	<0.002	0.986	<0.003	0.018	0.835	0.022
3	*UTP11L*	1	34	<0.002	0.828	<0.003	0.006	1.168	0.005
4	*C1orf174*	1	36	<0.002	2.473	<0.002	<0.002	2.049	<0.002
5	*KRTAP23-1*	21	2	<0.002	0.955	<0.003	<0.002	0.586	<0.004
6	*ZNF14*	19	49	<0.002	0.625	<0.004	0.012	0.788	0.015
7	*LOC101926911*	15	28	<0.002	0.526	<0.004	0.008	0.856	0.009
8	*SGSH*	17	45	0.002	1.839	0.001	0.004	0.842	0.005
9	*UROD*	1	6	0.002	1.523	0.001	0.040	1.870	0.021
10	*HES2*	1	13	0.002	0.858	0.002	0.016	0.694	0.023
11	*CHRNB1*	17	50	0.008	2.413	0.003	0.022	1.422	0.015
12	*ZBTB47*	3	42	0.004	1.106	0.004	0.076	1.465	0.052
13	*MIR4467*	7	2	0.004	0.879	0.005	0.006	0.848	0.007
14	*GTF3A*	13	31	0.006	0.918	0.007	0.002	1.178	0.002
15	*HMGB4*	1	20	0.012	1.593	0.008	0.062	1.248	0.050
16	*TEKT2*	1	27	0.012	1.481	0.008	0.006	1.782	0.003
17	*COX8A*	11	12	0.006	0.722	0.008	0.006	1.043	0.006
18	*MED29*	19	27	0.010	1.202	0.008	0.026	0.637	0.041
19	*C11orf21*	11	32	0.008	0.951	0.008	0.038	1.172	0.032
20	*PRAMEF17*	1	5	0.014	1.656	0.008	0.054	0.721	0.075

*Chr* chromosome, *p*_s_^*^ weight adjusted *p* value for SBP, *p value*_*s*_ *p* value for SBP, *# RVs* number of rare variants identified in the gene, *W*_*s*_ weight for SBP

Subscript D represents statistics for DBP

We performed gene-set enrichment analysis for these 153 significant genes using GO biological process categories. The top 15 enriched gene sets with more than ten genes are reported in Table 2 with *p* value of less than 0.05. The results suggest that these reported 153 genes are involved in the regulation of blood pressure, and blood vessel size pathways (*p* value <0.03). Interestingly, these 153 blood pressure–associated genes are also significantly involved in sensory perception of sound. Hypertension has been clinically observed to be correlated with hearing loss [12]. The result could suggest genetic basis for the correlation between hypertension and hearing function. However, further study is needed to validate the findings in this study.

**Table 2:** Enriched GO biological processes (*p* value <0.05) for the top 153 blood pressure–associated genes

	GOBPID	Count	Size	Term	*p* Value
1	GO:0007600	10	107	Sensory perception	0.0031
2	GO:0050873	3	12	Brown fat cell differentiation	0.0069
3	GO:0007605	4	26	Sensory perception of sound	0.0109
4	GO:0048869	26	495	Cellular developmental process	0.0123
5	GO:0003013	6	57	Circulatory system process	0.0123
6	GO:0042981	15	239	Regulation of apoptotic process	0.0142
7	GO:0012501	17	288	Programmed cell death	0.0161
8	GO:0008544	7	79	Epidermis development	0.0173
9	GO:0031424	4	30	Keratinization	0.0180
10	GO:0010941	15	246	Regulation of cell death	0.0182
11	GO:0007369	3	18	Gastrulation	0.0220
12	GO:0045638	3	18	Negative regulation of myeloid cell differentiation	0.0220
13	GO:0050880	3	19	Regulation of blood vessel size	0.0255
14	GO:0008217	3	20	Regulation of blood pressure	0.0277
15	GO:0016265	17	312	Death	0.0331

*GOBPID* GO biological process ID

## Discussion

Many of the genes reported in Table 1 achieve smaller *p* values after the weight adjustment procedure in this analysis. In Table 1, we listed genes with weight-adjusted *p* values of less than 0.05. Multiple comparisons, such as Bonferroni threshold, could be adapted and applied using the weight-adjusted *p* values. In this analysis, the threshold is 0.05/6118 ≅ 8 × 10^− 6^ and none of the genes reported in Table 1 exceeded this stringent threshold value. In addition, we chose the Seq-aSum-VS test to obtain a *p* value for each gene in this paper; other approaches, such as the sequence kernel association test (SKAT) [5], can also be used to obtain the *p* value for each gene and the weighting scheme proposed in this study can then be used to calculated weight-adjusted *p* values. To obtain ${w}_{g_i{E}_j}$, we summed the total number of rare variants across all the loci in a gene in equation (1) by assuming all the rare variants have similar effects on *E*_*j*_. An alternative approach to calculate ${w}_{g_i{E}_j}$ is to replace equation (1) by the test statistic reported Seq-aSum-VS test while treating gene expression as the outcome. The alternative approach does not assume that all the rare variants have the same effects on *E*_*j*_; however, the alternative approach might be more computationally intensive.

In this analysis, we did not consider genes with more than 50 rare variants. In a gene with a large number of rare variants, many of the variants might be null-variants, which will cause the estimated effects for genes with large numbers of rare variants to be diluted. For gene with a large number of rare variants, we suggest to use a moving window approach and only consider a feasible number of rare variants in a window.

Furthermore, for data collected in a case–control design, our proposed approach is easily modified by logistic regression and applied. The weighting scheme proposed in this study is also usable when only a subset of the individuals have gene expression measurements available. It is also modifiable for when *SNP*, gene expression data and gene expression, phenotype data are from two different sets of cohorts, instead of paired gene expression and GWAS data from the same cohort. However, paired gene expression and GWAS data from the same cohort might be preferable, as the data will have increased power to detect the causative relationship *(SNP→E→P)* but not the reactive relationship *(SNP→P→E)* based on the simulation study described in [8].

In the data analysis, we observed genes with small *p* values without evidence of gene expression association. It is biologically possible that genetic variants could cause phenotypic changes without altering gene expression level. Thus in practice, we suggest to pursue genes with either (a) small *p* values from Seq-aSum-VS test or (b) small weight-adjusted *p* values.

## Conclusions

In this paper, we proposed a novel approach to incorporate gene expression information into rare variant association analysis. Using the weight-adjustment approach, this method upweights the genes that contribute to phenotype-associated gene expression and downweights others. This weight-adjustment approach is expected to boost the power of association analysis by incorporating additional genomic information while keeping the FWER controlled at a nominal level. Both simulation studies and experimental findings reported in Li et al. [7] and Ho et al. [8] support the expected gain in power through the weight-adjustment procedure.
